# Miscellanea Medica

**Published:** 1861-07

**Authors:** 


### ART. X.—MISCELLANEA MEDICA.

### I. MY LORD BERKELEY'S COPPER NOSE.

*Henry, Nineteenth Lord Berkeley*:—"In the second of Queen Elizabeth [1559], having in March extremely heated himself by chasing on foot a tame deare in Yate Park, with the violence whereof he fell into an immoderate bleeding of the nose; for the stay whereof, by the ill counsel of some about him, he dipt his whole face into a basin of cold water, whereby that flush and fullness of his nose, which forthwith arose, could never be remedyed, though for present help he had physicians in a few days from London, and for better help came thither himself not long after to have the advice of the whole college, and lodged with his mother at her house in Shoe-lane."*

### II. A MEDICAL BET.

*Catherine, Lady Berkeley*:—"Being, in the sixteenth of Elizabeth, the mother of three daughters, and almost without hope of more children, especially of a son, which she, for the continuance of her house and her husband's name much desired, extreamly grieving that the male line of this antient family should end in her default, as she accompted it, she acquainted Mr. Francis Aylworth therewith, then of Kington Magna in Warwickshire, a little old werish man, but an excellent well-read practized chirurgist and physician, and for many years a gentleman lodged in their house. He gave her hope of conception, yea of a son, if she and her lord would for a few months be ruled by him. This, in a private conference between these three, was agreed upon, and promised to be observed.

'Children are given to men;
It's God that giveth them.'

She conceived, and in one year after this communication brought forth a son called Thomas, father of the Lord George, of whom I am next to write, to her unspeakable comfort, but never conceived after; what time Mr. Aylworth told me this story, about ten years after, at Callowdon, which I have at second hand heard also, that the lord hath privately told to some others. He added, that some months, or thereabouts, before her time of delivery, she sent for him, and kept him with her; and he (out of what observation I know not) being confident she went with a son,

* Fosbroke's *Berkeley Manuscripts*, p. 189.

offered to wage with her 10*l.* to 30*l.* that soe it was. She accepted the offer, most willing, no doubt, to loose, had the wager been thirty hundred. As soone as she was delivered, and understood it was a son, the first word she spoke was,—'Carry Aylworth his thirty pound ;' which purposely she had layd ready in gold in her chamber. This being the eleaventh of July, *anno* 1575. She also prevailed with her husband to sell him the said manor of Kington Magna in September following for 520*l.*, which he then held on lease for years."*

### III. MEDICAL PRACTICE, SIXTEENTH CENTURY :—EMPEROR CHARLES V.

"The Emperor's [Charles V.] stomach was this last week very much swoln, and he in great feebleness. The Queen, perceiving that pills made of soldanella, an herb that comes out of Italy, had done Monsieur der Ruellp good, purging his stomach of an incredible deal of water, and other raw and gross matter, willed Dr. Cornelius to break the matter to the Emperor, and to see whether his mind would serve him to take the same purgation. The Emperor agreed to it, and at four o'clock the next morning took it; which did so work his stomach, so purge him, that (saving your honours) he that did carry out that that came from him did faint by the way, and had much ado to keep himself on his feet, so much did the savour turn his stomach. It wrought on him nine times, besides twice upwards. We had not known of this, but J. Morysine, saving none would allow, have had need of Vesalius these five or six days, who, amongst other things, told me the Queen and Cornelius did utterly despair of his life. The Emperor, as he saith, is now as glad that he took it as the Queen and Cornelius were sorry that ever they consented to give it unto him. The physician doubts much the Emperor's recovery; but he has a body so able to deceive physicians, and so able to live upon small strength, that till he be gone indeed we will think he hath still to tarry a little while; for, seeing the purgation did him no more harm, it must needs be that it did him much good. The Emperor's apothecary told Ascham that his Majesty is very well amended, and will change his lodging out of the palace into his park garden, and will also shortly come abroad."†

---

* Fosbroke's *Berkeley Manuscripts*, p. 206.

† Sir Richard Morysine and Sir Thomas Chamberlayne to the Privy Council, from Brussels, 4th April, 1553. Lodge's *Illustrations of British History,* vol. i. p. 208, 2nd ed. 8vo.

### IV. CUTTING FOR THE STONE, 1608.

"Mr. Harry Willoughby, well known to your lordship, on Monday last, being (the 27th of this May) in the Old Bailey, at a barber's house (which barber cut up Mr. Kattrel when he was dead, thereby,) one Molenes, a man about thirty years, was cut for the stone in the bladder; and the bladder opened, and his instrument put in, and had ten pulls before he could get it. The stone is rough on both the sides; it is flat, and then round, like a flat bowl; almost as big as the ball of my hand. It seems he endured extreme pain with great impatience; yet this day, being Thursday, and the 30th of May [1608], he is very well, and like to recover."*

### V. EPIDEMIC CATARRH OF 1580.

"We have here in London, and at the Court, a new strange sickness. It does grieve men in the head, and with a stitch over the stomach. Few do die thereof, and yet many are infected. I do hear it credibly reported that forty students of Lincoln's Inn were taken with the said malady within the space of twenty-four hours. At the Court, the Lady Lincoln, the Lady Howard, the Lady Stafford, the Lady Leighton, are at this instant troubled therewithal. The Lord Lumley's sick there, and many of the inferior sort. Some say the Lord Chamberlain is sick at Newhall."†

### PLAGUE AT FULHAM, 1630—66. EXTRACTS FROM Y^E CHURCHWARDEN'S ACCOUNTS.

| | | | | |
|---|---|---|---|---|
| 1630. | An asseasement made the xxiij^rd daie of May, 1630, for further reliefe of y^e poore there, on Fulham side, for this present year, there being great cause, therefore, by reason of y^e poore's necessities in these times of scarcitie. Total received . . . . . | £3 | 6 | 0 |
| 1636. | An asseasement made this sixth daie of September, 1636, for the reliefe of the poor on Fulham side, to remaine in stocke, according to his Matie's order, in case the infection of the plague should be. Summe totall of this . . | 15 | 15 | 0 |

* Alexander Ratcliffe to the Earl of Shrewsbury. Lodge's *Illustrations of British History*, vol. iii. p. 235, 8vo edition.

† Thomas Bawldwin to the Earl of Shrewsbury, 1 July, 1580. Ibid. vol. ii. p. 175, 2nd edition, 8vo.

| | | | | |
|---|---|---|---|---|
| 1639. | Item, paid Osborne for two daies' warding at Lady Grivill's | £0 | 2 | 4 |
| | Paid Henry Young for watching and warding at the same place | 0 | 4 | 3 |
| | Paid Young, for warding three weeks and five days | 1 | 8 | 0 |
| | Paid Kelly for watching two nights | 0 | 2 | 0 |
| | Paid Nicholls for warding | 0 | 0 | 6 |
| | Paid Elizabeth Jones and widow Payne for being at the Lady Grivill's | 1 | 2 | 0 |
| | Paid for Shyercraft's dyet being shut upp | 0 | 11 | 9 |
| | Paid Gringle for Wood's wife being shut upp | 0 | 15 | 0 |
| | Paid for beefe for 3 visited houses | 0 | 7 | 1 |
| | Given to Goodwife Lake in her sickness, and for her keepe | 0 | 10 | 0 |
| | Paid for a sicke woman at the brickills, and for her keeper, the Easter daie last | 1 | 15 | 0 |
| 1640. | Item, to the bearers that came from London, and a deal board to bear the corpses to churche | 0 | 11 | 4 |
| | Item, to Goodman Tucker, his house being suspected to be visited | 0 | 1 | 0 |
| | Item, for the relief of Fuller's house from the 13th of December, 1640, to the 1st of Februarie following | 2 | 13 | 4 |
| | Item, for the buriall of Fuller | 0 | 2 | 0 |
| 1640. | Item, paid to James Francis Smyth, for a bar of iron, wht. 9½lb. at 3*d.* per pound, to close upp Powell's house door | 0 | 2 | 4½ |
| | Item, for Brade and his man's labour, to sett on the barr | 0 | 0 | 6 |
| | Item, to Goodman Burr for one week's wardinge | 0 | 5 | 0 |
| | ,, Goodman Osborne for wardinge | 3 | 0 | 8 |
| | Item, for bushel of coles for y[e] visit-house | 0 | 1 | 3 |
| | Item, for the reliefe of Elizabeth Joans, being shut upp in a visited house at Mansion's Green from the 15th of April, 1641, to the 24th May | 0 | 16 | 0 |
| | Item, for a trusse of strawe for her to lie on | 0 | 0 | 3 |
| | Item, to Goodwife Baker, in tyme of her weakness, before she got right to the hospital, where she died a pitiful creature | 0 | 6 | 0 |
| | Item, to Eliza Joanes, at several tymes, for her wardinge | 0 | 2 | 4 |
| | Item, to Humphrey Phillips, for his attendance in tyme of sickness | 0 | 4 | 6 |
| | Item, for his knell, a shroude for him, his grave makinge, and in expenses in the house by those that bare him to church | 0 | 6 | 2 |

| | | | |
|---|---|---|---|
| Item, for two trusses of strawe for him to lie on . | £0 | 0 | 6 |
| Item, paid to Danyell Carter for reliefe of himselfe, his wife, and children, before his house was shutt up of his sickness . . . . . . . | 0 | 19 | 6 |
| Item, to Goodman Shute in tyme of his sickness and his wife's before her death . . . . | 1 | 5 | 0 |
| Item, for a new spade to set him worke . . . . | 0 | 3 | 4 |
| Item, to Goodman Watkins in tyme of sickness before he dyed . . . . . . . . . . . | 0 | 10 | 6 |
| Item, to Mr. Cluett for the knell and grave makinge . . . . . . . . . . . . . | 0 | 0 | 6 |
| 1640. An asseasment made y^e last daye of February 1640, by John Bruton, churchwarden, and other the inhabitants of Fulham (on Fulham side) whose names are hereunto subscribed, and is for leaveying of 16*l.* 4s. 2d. forthwith, which saide sume is to make up y^e 8*l.* odde money, formerley asseased for reliefe of the visited houses, 24*l.* 17s. 2d., which said some Walter Sheldon, one of the overseers of the poore on Fulham side, hath disbursed and laid out for the reliefe of the visited houses, from the 26th of October, 1641, to the first of February nexte following . . . . . . . . . | 16 | 4 | 2 |
| 23 July, 1665. An asseasment for 60*l.* for the reliefe of the visited houses and families on Fulham side, and to discharge the tax laid upon y^e parish for relief of the visited in St. Giles.* | | | |

*Notes.*—" Among the employments which the plague itself furnished was that of watching the houses shut up by authority, the inhabitants of which were not allowed any kind of communication whatever but thro' the watchmen who relieved each other every 12 hours, and whose duty it was to procure provisions and other necessaries for the house they were appointed to guard."†

" The justices of Middlesex, by directions of the Secretaries of State, began the practice (of watching houses infected) in the parishes of St. Giles, St. Martin, and St. Clements, about the latter end of June, or the 1st of July; the Lord Mayor and Aldermen adopted a similar practice in the city and its liberties; and in a week or two afterwards, the magistrates of the Tower Hamlets ordered the same measure to be taken in the eastern parishes. Watchmen were appointed to guard the houses if not shut up both by night and day, and over every door thus closed a large red cross was marked, with this supplicatory sentence printed over it—'Lord have mercy upon us.' " ‡

* *An Historical and Topographical Account of Fulham, including the Hamlet of Hammersmith,* by T. Faulkner; pp. 141—144. London, 1813.

† *Brayley's London,* p. 400. ‡ Ibid. p. 389.

